# Measuring perinatal mental health risk

**DOI:** 10.1007/s00737-012-0297-8

**Published:** 2012-08-01

**Authors:** M. Johnson, V. Schmeid, S. J. Lupton, M.-P. Austin, S. M. Matthey, L. Kemp, T. Meade, A. E. Yeo

**Affiliations:** 1 Centre for Applied Nursing Research, South Western Sydney Local Health District/University of Western Sydney, Sydney, NSW Australia; 2 School of Nursing and Midwifery, University of Western Sydney, Sydney, NSW Australia; 3 School of Medicine, University of Western Sydney, Sydney, NSW Australia; 4 Perinatal and Women’s Mental Health Unit, St John of God Health Care, University of New South Wales, Sydney, NSW Australia; 5 Black Dog Institute, Prince of Wales Hospital, Sydney, NSW Australia; 6 School of Psychology, University of Sydney, Sydney, NSW Australia; 7 Infant, Child and Adolescent Mental Health Service, South West Sydney Local Health District, Sydney, NSW Australia; 8 Centre for Health Equity Training Research and Evaluation (CHETRE), part of the Centre for Primary Health Care and Equity, School of Public Health and Community Medicine, University of New South Wales, Liverpool Hospital, Locked Bag 7103, Liverpool BC, NSW 1871 Australia; 9 School of Psychology, University of Western Sydney, Sydney, NSW Australia; 10 Ingham Institute, South Western Sydney Local Health District, Sydney, NSW Australia

**Keywords:** Instruments, Psychosocial assessment, Perinatal, Mental health

## Abstract

The purpose of this review was to critically analyse existing tools to measure perinatal mental health risk and report on the psychometric properties of the various approaches using defined criteria. An initial literature search revealed 379 papers, from which 21 papers relating to ten instruments were included in the final review. A further four papers were identified from experts (one excluded) in the field. The psychometric properties of six multidimensional tools and/or criteria were assessed. None of the instruments met all of the requirements of the psychometric properties defined. Some had used large sample sizes but reported low positive predictive values (Antenatal Risk Questionnaire (ANRQ)) or insufficient information regarding their clinical performance (Antenatal Routine Psychosocial Assessment (ARPA)), while others had insufficient sample sizes (Antenatal Psychosocial Health Assessment Tool, Camberwell Assessment of Need—Mothers and Contextual Assessment of Maternity Experience). The ANRQ has fulfilled the requirements of this analysis more comprehensively than any other instrument examined based on the defined rating criteria. While it is desirable to recommend a tool for clinical practice, it is important that clinicians are made aware of their limitations. The ANRQ and ARPA represent multidimensional instruments commonly used within Australia, developed within large samples with either cutoff scores or numbers of risk factors related to service outcomes. Clinicians can use these tools, within the limitations presented here, to determine the need for further intervention or to refer women to mental health services. However, the effectiveness of routine perinatal psychosocial assessment continues to be debated, with further research required.

## Introduction

Internationally, it is reported that one in five women experience a perinatal mental health disorder within the first year after the birth of a baby, yet many women are neither diagnosed nor treated (Austin et al. 2008; Dennis and Allen 2008). Mental health problems such as depression, anxiety, drug and/or alcohol misuse and social problems such as domestic violence during the perinatal period are recognised as major public health issues (Buist et al. 2005) and are associated with poor outcomes for women and their children and partners (Murray et al. 2003). Intervention studies indicate that mental health problems during the perinatal period can be minimised if women and families engage in appropriate service (Armstrong et al. 2002; Kemp et al. 2011; Shaw et al. 2006).

There are known, identifiable risks for poorer maternal and/or infant and child outcomes. An Australian study of over 40,000 women (*beyondblue)* confirmed the importance of the following potential risk factors: mental health problems (prior and current), domestic violence, substance misuse, past history of abuse, anxiety, lack of support, separation, unemployment, lower socioeconomic status and a stressful pregnancy (Austin et al. 2008; Bilszta et al. 2008a; Buist et al. 2005). Pregnancy has been identified as a time of high risk for women experiencing domestic violence (Gartland et al. 2011), with increasing evidence that maternal anxiety and stress during pregnancy, such as that generated by domestic violence, can have a long-term negative impact on the foetus and infant (Glover and O’Connor 2006). The presence of multiple risk factors further increases the risk of poor maternal and infant outcomes, and adds to the complexity of prioritising support for these women throughout the perinatal period.

In response to this, governments in Australia and internationally are working to redesign and strengthen services provided to pregnant women, children and families ‘at risk’ of poor physical health and social and emotional outcomes. Major policy initiatives, such as Supporting Families Early in NSW Australia, incorporating the Safe Start Strategic Policy, emphasise that the perinatal period is a ‘window of opportunity’ to identify families who will benefit most from support and early intervention. Therefore, there has been a move towards routine assessment for psychosocial risk factors, including depression screening (beyondblue: the national depression initiative 2009).

A number of assessment tools exist to identify risk factors for postpartum emotional difficulties (Austin et al. 2008; Blackmore et al. 2006). Outcome studies of routine psychosocial assessment and depression screening have not yet identified a singularly agreed tool or measure to be used to identify those women who are most at risk of psychosocial or mental health concerns. A recent Cochrane review by Austin et al. (2008) showed that a concurrent measure of potential risk factors and emotional status in combination with raising the awareness of staff of other potential risk factors increases the detection of risk and mental ill health symptomatology in pregnancy. However, questions remain about the specificity and sensitivity of these tools and their capacity to detect women at risk for future depression or other perinatal mental health distress or disorder (Austin et al. 2008; Yelland et al. 2009). The aim of this review was to critically analyse existing tools to measure perinatal mental health risk and report on the psychometric properties of the various approaches using defined criteria.

## Methods

**Fig. 1:**
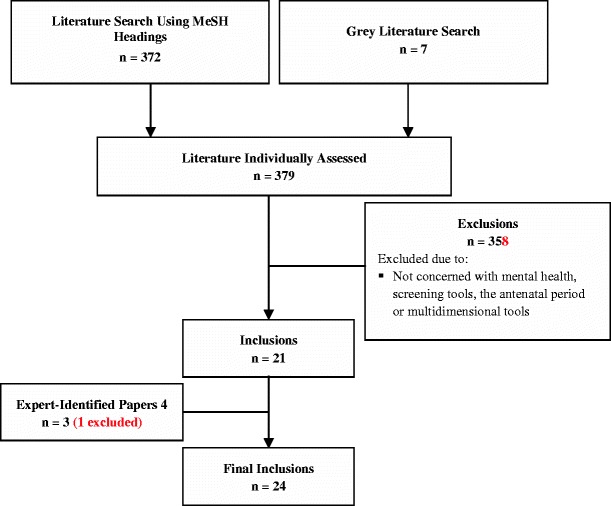
Systematic literature review inclusion and exclusion criteria

A systematic approach to searching the literature was initially undertaken to identify multidimensional tools used in perinatal mental health assessment, in both the cited and grey literature. Literature searches were conducted using Ovid Medline, CINAHL, Scopus, Ebsco, Psyclit, PubMed, Australian Federal and State Government websites and UK, US and New Zealand Government websites. The following MeSH terms were used: perinatal, postnatal, antenatal, female, assessment, psychological test/s, risk assessment, screening, reproducibility of results, questionnaires, instrumentation, depression, anxiety, mental health and mental disorders. Results were limited to literature published in English between 1990 and 2011. The 379 papers located were individually assessed. From this literature, 358 papers were judged as not representing instruments that measure the multidimensional nature of perinatal mental health risk. A further four papers were identified by experts in the field; one paper used the measurement of a single item and was not included in this review (Matthey et al. 2004; NSW Government Health 2010; Webster et al. 2000). Therefore, 24 papers (10 instruments) were finally included in this review (see Fig. 1).

In this review, we sought to identify and describe the multidimensional assessment tools, reflecting the multiple factors known to influence perinatal mental health. Several instruments examined only current emotional health, and thus are not part of this review, even though they may be used in conjunction with other tools measuring risk factors for predicting future emotional health. These include: the Pregnancy Depression Scale (Altshuler et al. 2008), the Edinburgh Depression Scale (EDS) (Bunevicius et al. 2009), the Beck Depression Inventory (Holcomb et al. 1996), the Postpartum Depression Predictors Inventory—Revised (Oppo et al. 2009), the Hospital Anxiety and Depression Scale (Jomeen and Martin 2004; Karimova and Martin 2003) and the Hamilton Rating Scale for Depression (Altshuler et al. 2008). Similarly, instruments that were limited to current anxiety, depression, somatic symptoms or social support (General Health Questionnaire (Kitamura et al. 1994), Hopkins Symptom Checklist—25 (Lee et al. 2008), Kessler 10 (Spies et al. 2009) and Maternal Social Support Scale (Webster et al. 2000)) which also did not capture the breadth of risk factors associated with future perinatal mental health risk were also excluded.

An initial step in this study was to define the psychometric properties and other characteristics required to critically analyse the various screening tools. Hammill et al. (1992) have developed a set of criteria that are consistent with key standards for psychological instruments as defined by the American Educational Research Association and the American Psychological Association (American Educational Research Association (AERA) American Psychological Association (APA) National Center on Measurement in Education (NCME) 1999). Several psychometric properties (reliability, validity, sensitivity, specificity, normative data and an overall scale rating) were included in the review criteria (Hammill et al. 1992) and are defined below.

### Reliability

The aspects of reliability relevant to this review included the inter-rater reliability (the consistency of the tool when used by different raters) and the test–retest reliability (the stability of the assessment tool when the tool is used by the same people on separate occasions) (Hammill et al. 1992; Taylor and Ranse 2011). Internal consistency (the ability of the items to assess the underlying construct) (Taylor and Ranse 2011), where applicable, has been considered.

### Validity

Several aspects of validity were considered in this review, including the assessment tool's content validity (the adequacy of coverage of the area being measured), the construct validity (the tool's ability to measure the construct in question) and the tool's criterion validity (the ability to distinguish amongst those who differ by a chosen criterion in their present status) (Hammill et al. 1992; Taylor and Ranse 2011).

### Sensitivity and specificity

Sensitivity and specificity of each assessment tool are included in this review, where available. The sensitivity of the assessment tool is the ability of the tool to correctly identify a ‘case’, meaning to correctly screen *in* a condition (Altman and Bland 1994a). Therefore, sensitivity is the rate of the tool in yielding ‘true positives’. Specificity is the assessment tool's ability to correctly identify non-cases, meaning to correctly screen *out* those without the condition (Altman and Bland 1994a). Therefore, specificity is the tool's rate of yielding ‘true negatives’. The positive predictive value (PPV) is the proportion of cases identified as at risk on the assessment tool who do develop emotional difficulties (‘true cases, or positives’) (Altman and Bland 1994b). The negative predictive value (NPV) is the proportion of cases identified as ‘not at risk’ on the assessment tool who do not proceed to develop emotional difficulties (‘true negatives’) (Altman and Bland 1994b; Hammill et al. 1992). The cutoff values for the tools, at which the sensitivity and specificity were calculated, are also presented where available.

### Normative data

Normative data typically refer to stratified data which are presented according to relevant variables. These show differences in scores related to age, gender, ethnicity and clinical diagnosis. Transformation of these raw scores into standard scores, percentile scores or cutoff scores is sometimes provided, and reporting of four to five demographic variables (including race, ethnicity, principal language spoken in the home, educational attainment, employment status and family income) is required according to Hammill et al. (1992). Where normative data were available, these are provided. In particular, aspects of normative data relating to age and ethnicity may be important in the context of perinatal mental health. Hammill et al. recommend samples sizes of 1,000 or more where there is variation in age, to ensure a normally distributed sample which is representative of the chosen population (Hammill et al. 1992).

### Overall scale rating

In order to assist readers in comparing the psychometric properties of the assessment tools, an overall rating was ascribed to each tool based on the model used by Hammill et al. (1992). This rating acts by breaking theoretical principles of measurement (such as statistical procedures, measurement issues and research designs as they relate to preferred practice in test construction) into standardised categories, so that useful, objective and unbiased judgements can be made about the technical characteristics of individual tools (Hammill et al. 1992). However, this rating is not intended to provide a definitive statement on a tool's psychometric rigour, which can alter with additional validation studies, but rather to offer a guide based on presently available evidence. The Hammill et al. (1992) scoring system awards the rating of the tool's reliability, validity and normative data as either ‘good’—A, ‘acceptable’—B or ‘unacceptable’—F. Ratings are based on criteria such as internal consistency, stability and test–retest reliability values, the current volume and quality of accumulated evidence regarding tool validity and the sample size, the diversity of demographic characteristics and the recency of the data collected. To obtain a final score, normative, reliability and validity ratings were combined to provide an overall rating of the scales as being either ‘highly recommended’ (two As), ‘recommended’ (As and/or Bs) or ‘not recommended’ (any Fs). The rating of the instruments using the Hammill et al. (1992) criteria was undertaken by investigators who had not contributed in any manner to the development or psychometric testing of any of the instruments presented.

## Results

Each of the identified multidimensional instruments or criteria is now described (also see Table 1).

**Table 1:** Reported validity and reliability for multidimensional perinatal mental health screening tools

Scale; reference	Total no. of items; no. of domains; scoring	Domains/criteria	Reliability and validity	Sensitivity (95 % CI) (Sn), Specificity (95 % CI) (Sp), negative predictive value (NPV), positive predictive value (PPV)	Sample size; normative data	Overall rating^a^
Multidimensional						
Antenatal Psychosocial Health Assessment Tool (ALPHA), Carroll et al. 2005	35; 4; 1–3	1. Family factors	OR (identifying a concern), 1.005 (95 % CI 0.6–1.7, *p* = 0.98); OR (identifying a high level of concern), 2.8 (95 % CI 0.7–11.7, *p* = 0.16); OR (family violence), 2.7 (95 % CI 1.1–6.9, *p* = 0.04)	Sn, not provided	227; yes	Not recommended with the present level of evidence due to: small sample size, sensitivity, specificity, positive and negative
		2. Maternal factors		Sp, not provided		
		3. Substance use		NPV, not provided		
		4. Family violence		PPV, not provided		
Antenatal Risk Questionnaire (ANRQ), Austin et al. 2011	12; 7; 1–5/6 or Y/N	1 Emotional support from subject's own mother in childhood	ROC AUC^b^, 0.69; 95 % CI = 0.61–0.77 (acceptable). Developed by a panel of experts, based on past reviews of postnatal depression risk factors, and on face and construct validity of these factors. The cutoff was based on ‘known groups’ using a diagnostic interview on women with high depression scores or items identifying distress.	At the most clinically relevant cutoff of 23 (out of a possible 62):	1,196; no	Not recommended with the present level of evidence. Due to: normative scores are not provided
		2 Past history of depressed mood or mental illness and treatment received		Sn, 0.62		
		3 Perceived level of support available following the birth of the baby		Sp, 0.64		
		4 Partner emotional support		PPV, 0.30		
		5 Life stresses in previous 12 months		NPV, 0.87		
		6 Personality style (anxious or perfectionistic traits)				
		7 History of abuse (emotional, physical and sexual)				
Australian Routine Psychosocial Assessment (ARPA), Matthey et al. 2004	12; 7; 1–5	1 Support	Face/content validity of items derived from existing known psychosocial risk factors. ‘Known groups’ validation was presented. No reliability/stability testing of items was reported.	Sn, not provided	2,167; no	Not recommended with the present level of evidence. Due to: normative scores, sensitivity, specificity, negative and positive predictive values not provided
		2 Stressors		Sp, not provided		
		3 Personality		NPV, not provided		
		4 Mental health		PPV, not provided		
		5 Childhood abuse				
		6 Family violence				
		7 Current mood measured by the Edinburgh Depression Scale [EDS]				
Camberwell Assessment of Need—Mothers (CAN-M), Howard et al. 2007	26; 26; 1–3	1 Accommodation	Calculated using the total number of unmet needs: Inter-rater reliability = 0.93 (service users), 0.83 (staff); test–retest reliability = 0.91 (service users), 0.85 (staff); content validity, expert reviewed; consensual validity, expert reviewed; concurrent validity = compared with the GAF-S (−0.36) and GAF-D (−0.52), using total summary scores	Sn, not provided	72; yes	Not recommended with the present level of evidence. Due to: small sample size, sensitivity, specificity, negative and positive predictive values not provided.
		2 Food		Sp, not provided		
		3 Looking after the home		NPV, not provided		
		4 Self care		PPV, not provided		
		5 Daytime activities				
		6 General physical health				
		7 Pregnancy care				
		8 Sleep				
		9 Psychotic symptoms				
		10 Psychological distress				
		11 Information				
		12 Safety to self				
		13 Safety to child and others				
		14 Substance misuse				
		15 Company				
		16 Intimate relationships				
		17 Sexual health				
		18 Violence and abuse				
		19 Practical demands of childcare				
		20 Emotional demands of childcare				
		21 Basic education				
		22 Telephone				
		23 Transport				
		24 Budgeting				
		25 Benefits				
		26 Language, culture and religion				
Contextual Assessment of Maternity Experience (CAME), Bernazzani et al. (2005)	4; 3; 1–4	1 Recent life adversity or stressors	Prospective study:	Sn, not provided	85; yes	Not recommended with the present level of evidence. Due to: small sample size, sensitivity, specificity, negative and positive predictive values were not reported.
		2 Quality of social support and key relationships including partner relationship	Domain 1	Sp, not provided		
		3 Maternal feelings towards pregnancy, motherhood and the baby	Predictive validity: RR, 1.57 (95 % CI 1.06–2.33)	NPV, not provided		
			Domain 2	PPV, not provided		
			Internal consistency: *α* = 0.86, *α* = 0.81			
			Concurrent validity: compared to measurement using EPDS			
			Domain 3			
			Internal consistency (maternal feelings towards pregnancy): *α* = 0.86, *α* = 0.82, *α* = 0.82			
			Internal consistency (maternal feelings towards motherhood and the baby): *α* = 0.87			
			Concurrent validity: compared to measurement using EPDS			
			Retrospective study:			
			Domain 2			
			Internal consistency: *α* = 0.81, *α* = 0.80			
			Concurrent validity: compared to measurement using EPDS			
			Domain 3			
			Internal consistency (maternal feelings towards pregnancy): *α* = 0.91, *α* = 0.94, *α* = 0.95			
			Internal consistency (maternal feelings towards motherhood and the baby): *α* = 0.85			
			Concurrent validity: compared to measurement using EPDS. For maternal feelings towards motherhood and the baby the concurrent validity was modest, and three overall indices were used in further analysis			
Pregnancy Risk Questionnaire (PRQ), Austin et al. 2005	21; 12; 1–6	1 Mother's attitude to her pregnancy	Area under the curve (AUC)^b^, 0.788 (95 % CI, 0.727–0.848). Compared to EDS. The AUC between the PRQ and the EDS were significantly different (0.788 and 0.659, respectively, *p* < 0.001). At the clinically relevant cutoff of 46 (the maximum *κ*), the odds that a woman scoring more than 46 and being a case was 9.18 times greater than for a woman scoring less 46 (OR, 9.18). Developed by a panel of experts, based on past reviews of postnatal depression risk factors, and on face and construct validity of these factors.	Sn, 0.44	1,296; yes	Not recommended with the present level of evidence. Low PPV
		2 Mother's experience of parenting in childhood		Sp, 0.92		
		3 History of physical or sexual abuse		NPV, 0.968		
		4 History of depression		PPV, 0.235 (at maximum *κ*)		
		5 Impact of depression on psychosocial function				
		6 Whether treatment was sought or recommended				
		7 Presence of emotional support from partner and mother				
		8 Presence of emotional support from partner and mother				
		9 Presence of stressors during pregnancy				
		10 Trait anxiety				
		11 Obsessional traits				
		12 Self-esteem				

^a^In order to assist readers in comparing the psychometric properties of the screening tools, an overall rating was ascribed to each tool based on the model used by Hammill et al. (1992). This rating is not intended to provide a definitive statement on a tool’s psychometric rigor, which can alter with additional validation studies but rather to offer a guide based upon presently available evidence. The Hammill et al. scoring system awards the rating of a tool's reliability, validity and normative data as either ‘good’—A, ‘acceptable’—B or ‘unacceptable’—F. Ratings are based on criteria such as internal consistency, stability and test–retest reliability values, the current volume and quality of accumulated evidence regarding tool validity and the size, diversity of demographic characteristics and recency of the normative sample. To obtain a final score, normative, reliability and validity ratings were combined to provide an overall rating of the scales as being either ‘highly recommended’ (two As), ‘recommended’ (As and/or Bs) or ‘not recommended’ (any Fs) {Hammill 1992, #381}

^b^Receiver operating characteristic (ROC) and area under the curve (AUC): the area under the ROC curve analysis provides an indication of a particular scale's diagnostic ability to discriminate between those with and without a particular diagnosis. The AUC values range from 0.5 to 1.0, where a value of 0.5 indicates that the scale is performing at a chance level, and 1.0 indicates perfect discrimination. There is no agreed standard for interpreting the significance of the AUC statistics. However, it has been suggested that values between 0.5 and 0.70 represent a scale with low accuracy, values between 0.70 and 0.90 are indicative of a useful screening scale and a value of 0.90 and above is indicative of a highly accurate screening scale with a perfect ability to identify those with the target diagnosis {Austin et al. 2005, #389}

### Antenatal psychosocial health assessment tool

The Antenatal Psychosocial Health Assessment (ALPHA) tool was developed by a multidisciplinary team of general practitioners, obstetricians, midwives and nurses in Canada (Reid et al. 1998). This tool uses 35 questions to identify antenatal psychosocial risk factors that would lead to poor postnatal psychosocial outcomes. These risk factors are associated with woman abuse, child abuse, postpartum depression and couple dysfunction, and the risk factors are further grouped into four categories: family factors, maternal factors, substance use and family violence. Questions are scored using a three-point tick-box system of ‘low’, ‘some’ and ‘high’. A study by Blackmore et al. (2006) amongst 227 antenatal women detected 38 % of probable cases of depression amongst women using the ALPHA when compared to the Edinburgh Postnatal Depression Scale (EPDS) used here as the standard in clinical use (Carroll et al. 2005; Blackmore et al. 2006). When compared to the control group, which did not receive the ALPHA questionnaire, the only category to show a significantly different odds ratio was for family violence (OR 2.7, 95 % CI 1.1–6.9). The categories of family and maternal factors did not reach statistical significance. Due to the small sample size of this study and the unknown sensitivity, specificity, positive and negative predictive values, this assessment tool is currently rated as not recommended.

### Antenatal risk questionnaire

The Antenatal Risk Questionnaire (ANRQ) was developed by Austin et al. (2011) in consultation with midwives and mental health care professionals working in a large maternity hospital. It consists of 12 items, which were selected from the original 23 Pregnancy Risk Questionnaire (PRQ) items (see below). This tool assesses the following psychosocial risk domains: emotional support from subject's own mother in childhood, past history of depressed mood or mental illness and treatment received, perceived level of support available following the birth of the baby, partner emotional support, life stresses in the previous 12 months, personality style (anxious or perfectionistic traits) and history of abuse (emotional, physical and sexual). It is scored using a combination of categorical and continuous data, with a possible maximum score of 62 and minimum score of 5. It was rated against the Composite International Diagnostic Interview (CIDI) diagnosis. The receiver operating characteristic area under the curve was 0.69 (acceptable) at the most clinically relevant cutoff of 23. At this cutoff, the sensitivity was 0.62, the specificity was 0.64, the PPV was 0.30 (low) and the NPV was 0.87. The acceptability of the ANRQ was high amongst both pregnant women and midwives (Austin et al. 2011). The presence of a cutoff score can greatly help clinicians in grading mental health risk magnitude. However, as this study did not comment on the demographics (4 to 5 required) of the study population (normative data), at present the ANRQ is rated as not recommended.

### Australian routine psychosocial assessment

The Australian Routine Psychosocial Assessment (ARPA) was developed by Matthey et al. (2004) from known psychosocial factors associated with parenthood. The assessment tool includes 12 questions (support, stressors, personality, mental health, childhood abuse, family violence and current mood, as measured by the EDS). The EDS is self-administered initially and then followed by the 12 ARPA questions. Women (2,173 English-speaking) attending the antenatal clinic were assessed, representing 97 % of all women attending the clinic over a 12-month period. Face and content validity are evident as the assessment domains examine known mental health risk factors (face) and the authors tested the questions on women and modified items as required (Matthey et al. 2004). Further validity is explored through an association between the number of risks and the services taken, with a gradual rise from women with two mean risk factors (33 %) remaining in phone contact with clinicians to women with 2.9 (31 %) mean risk factors receiving one or more face-to-face contacts, and a small group of women (3.1 %) with 3.7 mean risk factors being contacted and referred elsewhere (Matthey et al. 2004), ‘known groups’ validation. Validity was further suggested through the demonstration of similar proportions of women presenting with a history of anxiety or depression and/or domestic violence as those recorded by other known study populations. In addition, both women from English-speaking- and non-English-speaking backgrounds reported the ARPA as acceptable. Sensitivity, specificity, PPV and NPV were not reported, as women who were classified as at risk from this assessment were then offered an intervention. No reliability data or testing were reported. This tool receives an overall rating of not recommended due to a lack of sensitivity, specificity and normative data.

### Camberwell assessment of need—mothers

This tool was developed to aid in the identification of mothers with severe mental illness, to allow prompt referral to appropriate services made available by the UK Department of Health. The Camberwell Assessment of Need—Mothers (CAN-M) was based upon the Camberwell Assessment of Need, which is used in the general (non-pregnant) population to identify those with severe mental illness (Howard et al. 2007). The CAN-M covers the 26 domains of accommodation, food, looking after the home, self care, daytime activities, general physical health, pregnancy care, sleep, psychotic symptoms, psychological distress, information, safety to self, safety to child and others, substance misuse, company, intimate relationships, sexual health, violence and abuse, practical demands of childcare, emotional demands of childcare, basic education, telephone, transport, budgeting, benefits, language, culture and religion. Domains were assessed on a five-point Likert scale of importance (ranging from ‘not at all’ to ‘essential’). Inter-rater reliability was excellent at 0.93 for service users and 0.83 for staff. Test–retest was acceptable at 0.91 and 0.85 for service users and staff, respectively. Content validity and consensual validity (obtained through agreement of the expert panel that this screening tool measured the target paradigms) were both judged as good, as the tool was reviewed by an expert panel. Concurrent validity was −0.36 when compared with the Global Assessment of Functioning—Symptomatology and −0.52 when compared to the Global Assessment of Functioning—Disability. However, as the sample size of this study was only 72, and sensitivity, specificity, PPV and NPV were not provided, this tool received an overall rating of not recommended.

### Contextual assessment of maternity experience

The Contextual Assessment of Maternity Experience (CAME) is designed to encompass the two types of vulnerability which contribute to the risk of mental illness: (1) environmental vulnerability, including lack of support and disadvantaged socioeconomic conditions and (2) psychological vulnerability, such as low self-esteem and helplessness. The three domains explored in this tool are: recent life adversity or stressors, the quality of social support and key relationships including partner relationship, and maternal feelings towards pregnancy, motherhood and the baby. The paper by Bernazzani et al. (2005) reported on a prospective and a retrospective study. In the prospective study, women with a history of depression were screened. The internal consistency was good at >0.80 for each of the domains. Concurrent validity was also good, as the CAME was measured against the EPDS. In the retrospective study, data from women with material deprivation were analysed. Internal consistency was again good, ranging from 0.80 to 0.95 for each of the three domains. Concurrent validity was judged as good for the first two domains but only as modest for the third domain. As the study by Bernazzani et al. (2005) involved a sample size of only 85, and sensitivity, specificity, PPV and NPV were not provided, the CAME is currently judged as having an overall rating of not recommended.

### Pregnancy risk questionnaire

The PRQ contains 18 antenatal items and three early postnatal items (Austin et al. 2005). The 12 domains include: the mother's attitude to her pregnancy, mother's experience of parenting in childhood, history of physical or sexual abuse, history of depression, impact of depression on psychosocial function, whether treatment was sought or recommended, presence of emotional support from partner and mother, presence of other supports, presence of stressors during pregnancy, trait anxiety, obsessional traits and self-esteem. A five-point Likert scale is used, from 1 ‘not at all’ to 5 ‘very much’. The PRQ was implemented in this study alongside the EDS in 1,296 women. The area under the curve was 0.79 (significantly different to the EDS score of 0.66), indicating that, as a tool, the PRQ has an adequate level of accuracy when false positives and false negatives are of equal importance. At this level, the PPV and the sensitivity were low, at 0.24 and 0.44, respectively, whereas the NPV and specificity were high, at 0.97 and 0.92, respectively. Although the PRQ examined a large, normally distributed cohort, this tool received an overall rating of not recommended due to the low PPV.

## Discussion

Risk factors for women's mental health disorders are well established and well validated (Austin et al. 2005; Bilszta et al. 2008a, b; Buist et al. 2005; Dennis and Chung-Lee 2006; Matthey et al. 2004; Murray et al. 2003). This study reviewed existing instruments designed to assess the risk of the development of perinatal mental health disorders. Six multidimensional assessment tools were located and analysed. Many of the tools base their questions on previously described mental health risk factors, although not all (Kitamura et al. 1994). These risk factors included a personal history of depression, the presence of domestic violence, the lack of support from a partner and negative personal childhood experiences (Austin et al. 2005, 2011; Bernazzani et al. 2005; Carroll et al. 2005; Howard et al. 2007).

The assessment tools located were assessed for psychometric properties and rated according to the defined criteria as well as specificity and sensitivity. Although this system made it possible to compare tools, a paucity of normative data and the small sample sizes reported in the literature were particularly problematic. Normative data provide important information on the performance or scores within a defined group for the instrument or criterion. Obtaining normative data is a function of sample size, as the larger the sample, the more likely it is to be representative of the population (Hammill et al. 1992). Therefore, in order to ensure a representative and normally distributed sample, studies of a population fewer than 1,000 women here received a score of not recommended. These two criteria (normative data and adequate sample size) were unmet by several tools, resulting in a rating of not recommended. This current lack of data may reflect the relatively new nature in many countries regarding regular assessment of women for psychosocial and mental health issues in the perinatal period. Further testing of several of these instruments or domains in larger samples is anticipated. All assessment tools received an overall rating of not recommended; however, the ANRQ has fulfilled the requirements of this analysis more comprehensively than any other instrument examined here based on the defined rating criteria.

The majority of instruments assessed psychosocial and mental health in both the antenatal and postnatal period (the ALPHA, CAME, CAN-M and PRQ), and several authors note the importance of this (Locicero et al. 1997). Other tools were only used during the antenatal period (the ARPA, ANRQ). For diagnosticians, the availability of specificity and sensitivity data in both time periods may be of considerable benefit within the reporting of the psychometric properties relating to these tools (NSW Department of Health 2009; NSW Government Health 2010).

For clinicians, issues of sensitivity, specificity and normative data are particularly relevant. For the ALPHA, no sensitivity or specificity data were provided, only normative data. For the ANRQ, Austin suggests that the low PPV and sensitivity reported may have been due to the low prevalence of depression in the sample, a delay between the administration of the psychosocial assessment tool antenatally (ANRQ) and the diagnostic gold standard postnatally (CIDI), and significant dropout rates in the study (Austin et al. 2011). A study that validates the ANRQ with a diagnostic interview proximal to the time of ANRQ administration will better assess its PPV and sensitivity. The ANRQ recommends a cutoff score, which can be very helpful to the inexperienced clinician. In the ARPA study, sensitivity and specificity were not reported. However, known groups validation was presented as a surrogate for specificity. With 2,173 women included in the study, this represents the largest study of risk factor assessment found in the literature. The CAME reported excellent internal consistency, sensitivity and specificity but in a very small sample size of 85 (Bernazzani et al. 2005) making the clinical application difficult to support. Sensitivity and specificity data were also absent in the CAN-M study, and as this study only involved 72 women, validation through a larger sample population is needed.

In contemporary practice in Australia, the multidimensional psychometric scales currently used include the ANRQ which is administered both antenatally and postnatally (the PNRQ) and the ARPA. Both the ANRQ and ARPA represent appropriate domains and items of identified psychosocial risk factors, which have been adopted within state policies in Australia for the identification of perinatal mental health risk (Austin et al. 2011).

We suggest that to undertake comprehensive psychosocial assessment there is a need to combine both a cross-sectional tool that will identify current depressive or anxiety symptoms, for example the EPDS, and a broader tool that will assess the psychosocial vulnerabilities (‘current’ and ‘past but not current’ symptoms) that the woman brings to the perinatal period. Such tools are complementary, and combining a unidimensional (e.g., EPDS) and multidimensional tool (e.g., the ANRQ, ARPA) in this way allows for both cross-sectional and contextual/longitudinal factors to be assessed. In addition, psychosocial assessment tools can benefit from having clinically meaningful scores that guide the primary health care clinician in their decisions around the referral and management of their clients. However, clinical judgement must always prevail, as these tools are only an adjunct and not a substitute for clinical assessment. Structured guidance surrounding the interpretation of scores, items and management would support clinical decisions made by primary health care providers.

From this review of assessment tools, it is evident that further research is needed with larger samples to determine the predictive value of psychosocial screening and assessment tools. However, in practice, clinicians need guidance in how to assess psychosocial health and make decisions about the level of support to offer a woman and her family. In Australia, the recently released clinical practice guidelines for depression and related disorders concluded that there is insufficient evidence to support or refute the use of a specific tool for assessing the risk for depression or related disorders in the antenatal period, stating ‘there is no evidence that…the use of these tools improves referral or relevant outcomes among women in the perinatal period’(beyondblue the national depression initiative 2011, p. 18). However, the guidelines noted that enquiry into certain psychosocial factors of a significant nature by clinicians is endorsed by other relevant clinical practice guidelines (British Columbia Perinatal Health Program 2003; Scottish Intercollegiate Guidelines Network 2012). These factors include a past history of mental health disorders, available support, current or past abuse/violence and current life events. The included domains of enquiry were also supported by the findings of a large Australian prospective study (Milgrom et al. 2008). In the new guidelines, emphasis is placed on adapting the questions to the individual situation and context. It is highlighted that ‘as with any consultation, the longer the discussion, the greater the rapport that is likely to develop between the woman and health professional’, increasing the probability that the woman will feel able to speak openly and therefore, increasing the likelihood that any psychosocial factors will be identified (Austin et al. 2011).

Although this review aimed to compare and contrast assessment tools in regard to definite constructs, this was very difficult to accomplish. Much data for each of the different scales were absent, which made comparison very difficult. Debate currently exists surrounding the Supporting Families policy and the Safe Start guidelines (including assessment criteria) within this policy (Yelland et al. 2009), as to whether this represents best practice for Australian hospitals, as suggested in the beyondblue guidelines. Further testing of the tools with clinical outcomes data within large samples may confirm the diagnostic capabilities of the tool and confirm aspects of the criteria.

Other issues that need to be considered in assessment tools are whether the tools are health professional administered, via a face-to-face interview with the woman, or self-administered via a questionnaire. The former method allows for immediate clarification or explanation of questions or responses and develops rapport between the woman and the health professional. However, the latter method may facilitate openness when responding to particularly sensitive questions (for example childhood abuse). Very few studies have explored the impact of the method of administration on the psychosocial assessment process. Further, much of the research has explored psychosocial assessment in relation to postnatal depression, rather than postnatal depression or anxiety (‘distress’), an area for further research. It is also possible that some risks, or combinations of risks, need to be weighted more than others, which requires further investigation.

Although the focus of this review is on assessment tools, there is now a need to evaluate the effectiveness of combining psychosocial assessment with integrated pathways to care. A recent randomised controlled trial of early postnatal screening using the EPDS and supportive counselling (for women scoring >12) demonstrated improved maternal mental health outcomes at 6 months for those receiving the intervention (as part of an integrated screening and management programme) compared to the usual care (Leung et al. 2010). In a meta-analysis of screening interventions for general depression, Pignone et al. (2002) also found that screening was beneficial, as long as it was integrated with clear pathways to care (Pignone et al. 2002). This area of research may yield more useful results than the simple assessment of the PPV or sensitivity and specificity of particular tools.

Although the approach by Hammill et al. (1992) has provided excellent criteria, these criteria focus only on technical adequacy (Glover and Albers 2007). We acknowledge that many other aspects of these tools could have been addressed including appropriateness for the intended use or usability (Glover and Albers 2007). However, Glover and others (American Educational Research Association (AERA) American Psychological Association (APA) National Center on Measurement in Education (NCME) 1999) support the appropriateness of these criteria (adequacy of norms, predictive validity (sensitivity, specificity, PPV and NPV), reliability and concurrent, and construct and content validity) as essential when developing instruments for psychological testing and diagnostic purposes.

## Conclusion

Both unidimensional and multidimensional instruments are used worldwide to screen for perinatal mental health problems during the antenatal and postnatal periods. Although no tool fulfilled all of the requirements using the defined criteria, several tools are currently used in practice, such as the ANRQ and ARPA within their limitations. Further research is currently being undertaken to address issues of sensitivity and specificity for these instruments while other instruments require studies using larger sample sizes and to present normative data. Tools that guide clinicians in the identification and management of mental health risk can aid in timely intervention, treatment and the minimization of suffering for women worldwide.
